# Supplementary material for: Alternative splicing and residual function potentially expand the therapeutic landscape of the CFTRdele2ins182 variant
Source: PLoS One. 2025 Sep 16;20(9):e0330974. doi: 10.1371/journal.pone.0330974 (PMC12440211; doi:10.1371/journal.pone.0330974)
Supplement: S1 Table — (PDF) [file pone.0330974.s001.pdf]

S1 Table. Conversion between Legacy names and HGVS nomenclature of the cited CFTR variants.

**Legacy name**	***HGVS***	**dbSNP ID**
	RefSeq NM_000492.4	
CFTRdele2ins182	c.54-5811_164+2186del8108ins182	-
186-13C>G	c.54-13C>G	rs397508749
1717-1G>A	c.1585-1G>A	rs76713772
1898+3A>G	c.1766+3A>G	rs397508298
	c.870-1113_870-1110del	rs397508809

HGVS, Human Genome Variation Society
