# Supplementary material for: Alternative splicing and residual function potentially expand the therapeutic landscape of the CFTRdele2ins182 variant
Source: PLoS One. 2025 Sep 16;20(9):e0330974. doi: 10.1371/journal.pone.0330974 (PMC12440211; doi:10.1371/journal.pone.0330974)
Supplement: S1 Fig — While the molecular characterization of the patient FI077 was in progress, three additional CF patients (donor IDs: FI066, GE202 and MI253) carrying the same complex genomic rearrangement CFTRdele2ins182 were recruited. The amplification and the sequencing of the RT-PCR products spanning the CFTRdele2ins182 showed, also in these patients, the same pattern of transcripts, with mRNAs lacking exon 2 and both exon 2 and 3, as schematically represented in A and confirmed by Sanger sequencing of the RT-PCR products form patient’s cDNA spanning CFTR exons 1–5 (B). (PDF) [file pone.0330974.s002.pdf]

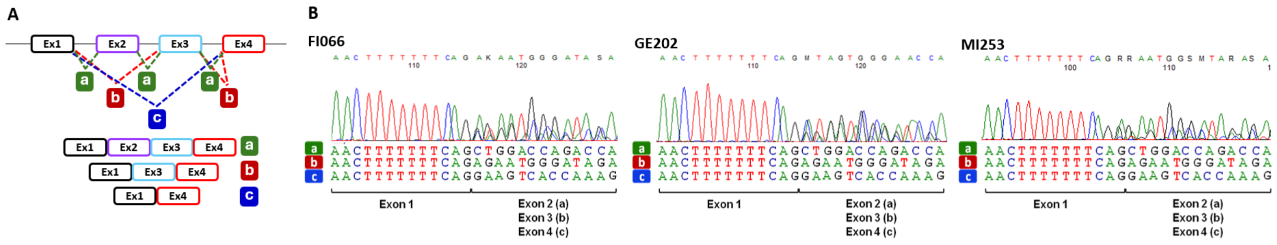

S1 Fig. The CFTRdele2ins182 variant induces an alternative splicing of exon 2 and 2/3 in primary HNEC from three unrelated CF patients carrying the variant. While the molecular characterization of the patient FI077 was in progress, three additional CF patients (donor IDs: FI066, GE202 and MI253) carrying the same complex genomic rearrangement CFTRdele2ins182 were recruited. The amplification and the sequencing of the RT-PCR products spanning the CFTRdele2ins182 showed, also in these patients, the same pattern of transcripts, with mRNAs lacking exon 2 and both exon 2 and 3, as schematically represented in **A** and confirmed by Sanger sequencing of the RT-PCR products form patient's cDNA spanning *CFTR* exons 1 to 5 (**B**).
