# Supplementary material for: Alternative splicing and residual function potentially expand the therapeutic landscape of the CFTRdele2ins182 variant
Source: PLoS One. 2025 Sep 16;20(9):e0330974. doi: 10.1371/journal.pone.0330974 (PMC12440211; doi:10.1371/journal.pone.0330974)
Supplement: S2 Fig — A. Scatter plot showing the overall abundance of CFTR transcripts in CF and control individuals measured by qPCR and normalized to β2microglobulin and HPRT housekeeping genes expressed as 2^-ΔCt. Each dot represents the mean CFTR transcripts overall abundance for each individual. For each subject, two biological replicates (n = 2) were analysed due to the limited availability of samples. B. Stacked column chart representing the relative expression of the different transcripts lacking exon 2 or exons 2 and 3 expressed as a percentage of the overall CFTR mRNA set as 100% for each CF sample. The analysis has been performed by qPCR on the RNA obtained from the differentiated epithelia of the three indicated CF samples, with oligonucleotide mapping in the different regions of the CFTR cDNA to quantify the overall and allele-specific abundance of the different transcripts. (PDF) [file pone.0330974.s003.pdf]

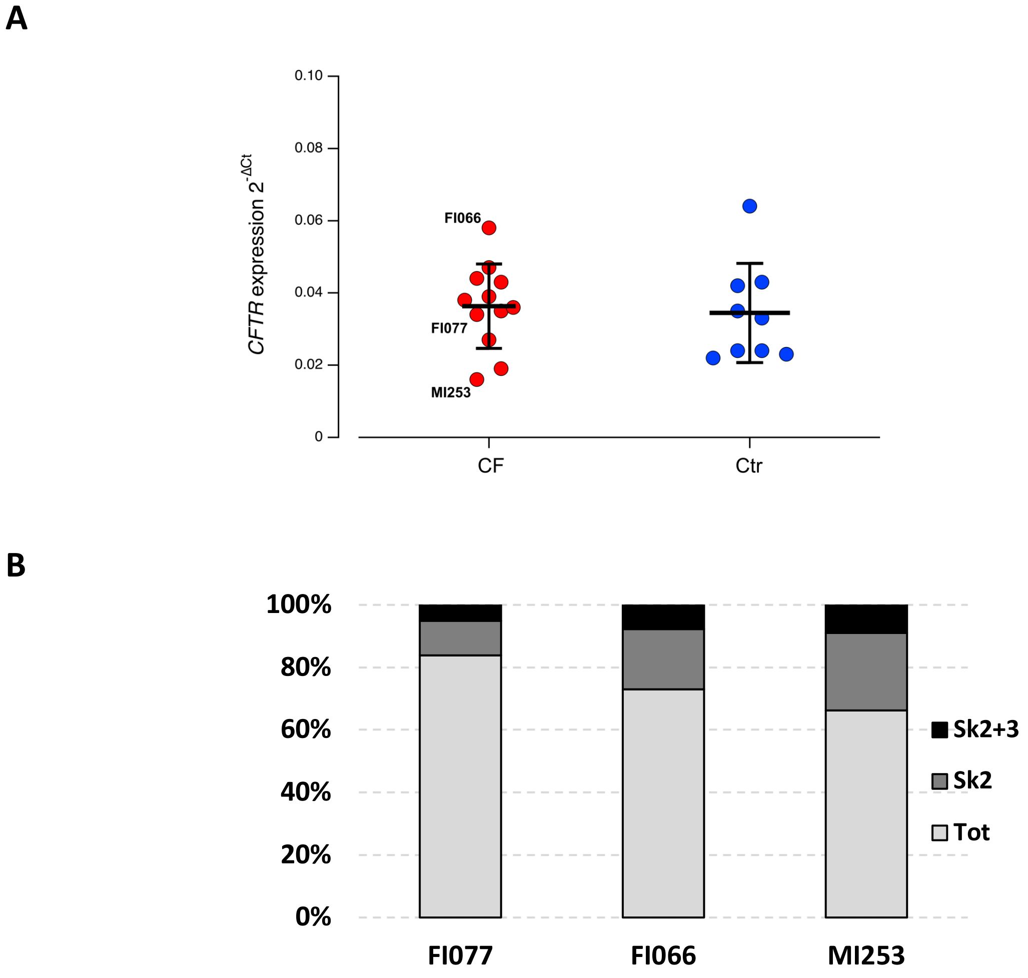

S2 Fig. Quantitative analysis of *CFTR* transcripts in nasal epithelial cells derived from FI077, FI066 and MI253 individuals by qPCR.

A. Scatter plot showing the overall abundance of *CFTR* transcripts in CF and control individuals measured by qPCR and normalized to β2microglobulin and HPRT housekeeping genes expressed as $2^{-\Delta Ct}$. Each dot represents the mean *CFTR* transcripts overall abundance for each individual. For each subject, two biological replicates (n = 2) were analysed due to the limited availability of samples. **B**. Stacked column chart representing the relative expression of the different transcripts lacking exon 2 or exons 2 and 3 expressed as a percentage of the overall CFTR mRNA set as 100% for each CF sample. The analysis has been performed by qPCR on the RNA obtained from the differentiated epithelia of the three indicated CF samples, with oligonucleotide mapping in the different regions of the CFTR cDNA to quantify the overall and allele-specific abundance of the different transcripts.
