# Supplementary material for: Alternative splicing and residual function potentially expand the therapeutic landscape of the CFTRdele2ins182 variant
Source: PLoS One. 2025 Sep 16;20(9):e0330974. doi: 10.1371/journal.pone.0330974 (PMC12440211; doi:10.1371/journal.pone.0330974)
Supplement: S1 Raw images — (PDF) [file pone.0330974.s004.pdf]

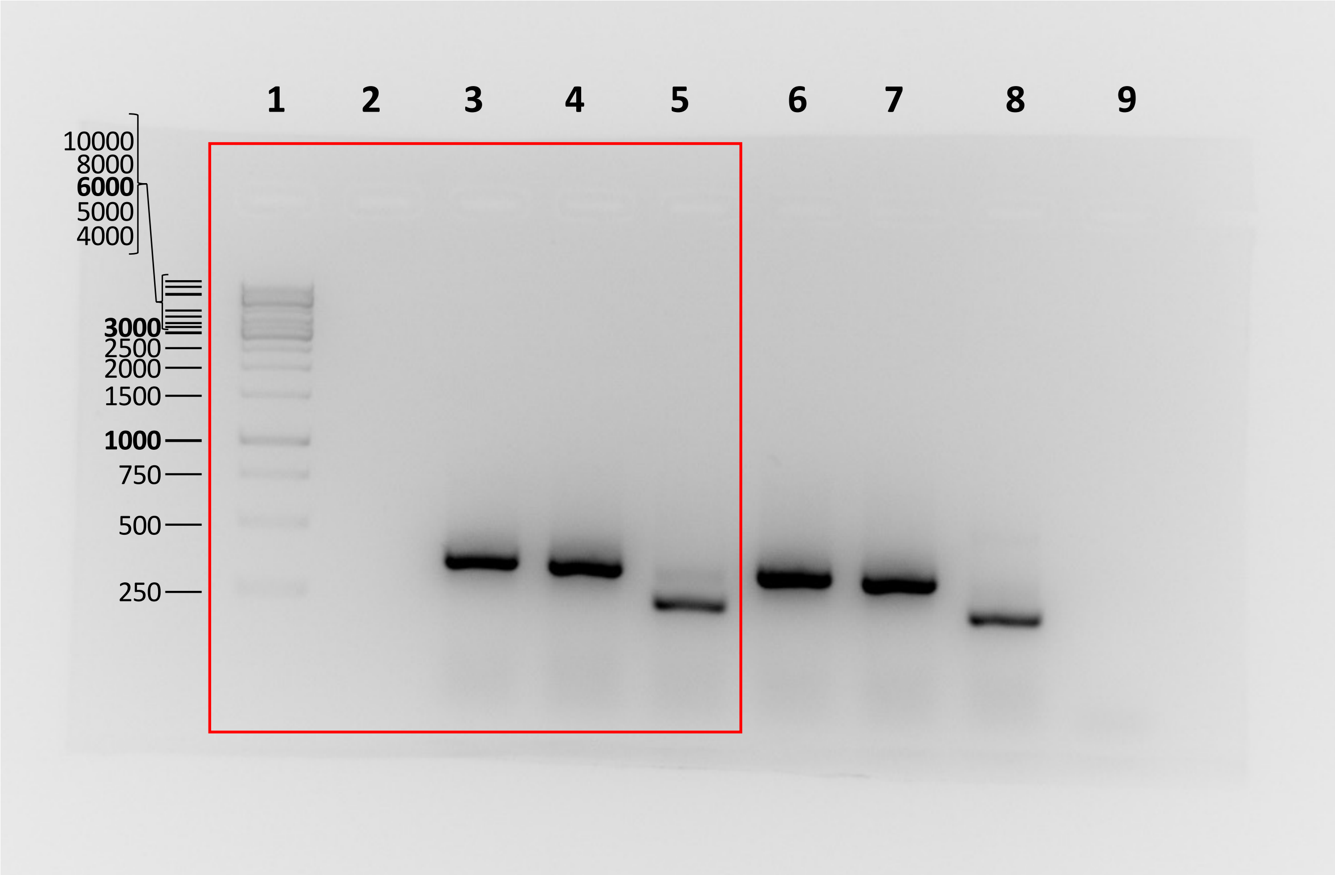

Uncropped agarose gel image used for the panel B of Figure 2: Minigene approach for the functional characterization of the 186-13C>G variant. The red rectangle indicates the image area selected for the figure. The image has been acquired with Molecular Imager Chemidoc XRS System (Bio-rad Laboratories Inc., Hercules, CA, United States**).**

Lanes: 1, Gene Ruler 1Kb molecular weight marker ladder (Fermentas); **2**, no template control; RT-PCR product obtained in cell transfected for 24 hours with the wild-type minigene (374 bp); **3**, RT-PCR product obtained in cell transfected for 24 hours with the mutated minigene (374 bp); **4**, RT-PCR product obtained in cell transfected for 24 hours with the empty pSPL3 splicing vector (263 bp); **6,** RT-PCR product obtained in cell transfected for 48 hours with the wild-type minigene (374 bp); **7**, RT-PCR product obtained in cell transfected for 48 hours with the mutated minigene (374 bp); **8**, RT-PCR product obtained in cell transfected for 48 hours with the empty pSPL3 splicing vector; **9**, no template control.
