# Supplementary material for: Alternative splicing and residual function potentially expand the therapeutic landscape of the CFTRdele2ins182 variant
Source: PLoS One. 2025 Sep 16;20(9):e0330974. doi: 10.1371/journal.pone.0330974 (PMC12440211; doi:10.1371/journal.pone.0330974)
Supplement: Attachment — Submitted filename: reply to comments.pdf [file pone.0330974.s006.pdf]

Editorial comments

1. Please ensure that your manuscript meets PLOS ONE's style requirements, including those for file naming. The PLOS ONE style templates can be found at https://journals.plos.org/plosone/s/file?id=wjVg/PLOSOne_formatting_sample_main_body.pdf and https://journals.plos.org/plosone/s/file?id=ba62/PLOSOne_formatting_sample_title_authors_affiliations.pdf

Reply: We have modified manuscript formatting and file naming to meet PLOS ONE's style requirements

2. Thank you for stating the following financial disclosure: "Fondazione per la Ricerca sulla Fibrosi Cistica grants n. FFC #3/2023, n.FFC #9/2019 and n. FFC #10/2021; Italian Ministry of Health [grant n. GR-2018–12367126, PNRR-MR1-2023-12378412] and through Cinque per mille (5Xmille 2019 grant 5M-2019-23680413) and Ricerca Corrente."

Please state what role the funders took in the study. If the funders had no role, please state: "The funders had no role in study design, data collection and analysis, decision to publish, or preparation of the manuscript." If this statement is not correct you must amend it as needed.

Please include this amended Role of Funder statement in your cover letter; we will change the online submission form on your behalf.

Reply: The requested statement has been added, and the change has been included in the Cover letter of the revised manuscript

3. PLOS ONE now requires that authors provide the original uncropped and unadjusted images underlying all blot or gel results reported in a submission's figures or Supporting Information files. This policy and the journal's other requirements for blot/gel reporting and figure preparation are described in detail at https://journals.plos.org/plosone/s/figures#loc-blot-and-gel-reporting-requirements and https://journals.plos.org/plosone/s/figures#loc-preparing-figures-from-image-files. When you submit your revised manuscript, please ensure that your figures adhere fully to these guidelines and provide the original underlying images for all blot or gel data reported in your submission. See the following link for instructions on providing the original image data: https://journals.plos.org/plosone/s/figures#loc-original-images-for-blots-and-gels.

In your cover letter, please note whether your blot/gel image data are in Supporting Information or posted at a public data repository, provide the repository URL if relevant, and provide specific details as to which raw blot/gel images, if any, are not available. Email us at plosone@plos.org if you have any questions.

Reply: The document including the uncropped gel image was already provided at the first submission. However, thanks to the editorial comment we realized that the provided uncropped image was not properly annotated. We have thus resolved this issue by providing the document with the requested details (S1_raw_image_REV).

4. We note that you have included the phrase "data not shown" in your manuscript. Unfortunately, this does not meet our data sharing requirements. PLOS does not permit references to inaccessible data. We require that authors provide all relevant data within the paper, Supporting Information files, or in an acceptable, public repository. Please add a citation to support this phrase or upload the data that corresponds with these findings to a stable repository (such as Figshare or Dryad) and provide and URLs, DOIs, or accession numbers that may be used to access these data. Or, if the data are not a core part of the research being presented in your study, we ask that you remove the phrase that refers to these data.

Reply: Following this comment, we are now presenting the relevant data as supplementary Figure S2.

5. Please ensure that you refer to Figure 4 in your text as, if accepted, production will need this reference to link the reader to the figure.

Reply: The legend to Figure 4 (page 16) has been maintained in the main text by mistake, as the corresponding figure has been moved to the Supplementary material as Figure S1. The numbering of Figures included in the main text is thus changed from 1 to 5. All the Figures are properly cited in the text.

6. If the reviewer comments include a recommendation to cite specific previously published works, please review and evaluate these publications to determine whether they are relevant and should be cited. There is no requirement to cite these works unless the editor has indicated otherwise.

Reply: No request to include citation of previous article has been raised by Reviewers.

7. Please review your reference list to ensure that it is complete and correct. If you have cited papers that have been retracted, please include the rationale for doing so in the manuscript text, or remove these references and replace them

with relevant current references. Any changes to the reference list should be mentioned in the rebuttal letter that accompanies your revised manuscript. If you need to cite a retracted article, indicate the article's retracted status in the References list and also include a citation and full reference for the retraction notice.
Reply: the list of references has been checked and is complete and correct, we did not cite any retracted articles.

Review Comments to the Author

Reviewer #1: This manuscript presents an analysis of rare CFTR mutations, with a particular focus on the complex allele [186-13C>G; 1898+3A>G] and the CFTRdele2ins182 variant, both of which have not been extensively characterized to date. The findings provide valuable insights into the CFTR mutation spectrum and their functional consequences.
Reply: We thank the reviewer for the positive comment.

While the data overall support the conclusions, several revisions are necessary prior to publication:
Figure quality: The resolution of the figures is generally low and should be improved to ensure data clarity and readability.
Reply: We apologize for the low quality of the figures. However, this was caused by the conversion of the high-quality tiff images (provided by us) into pdf during the uploading of the files to generate the single document used for reviewing purposes. The high-quality figures will be eventually used for publication if the manuscript is accepted.

Figure 5*:* If available, time-course data should be included to better illustrate the dynamics of the observed effects. Furthermore, appropriate statistical analyses should be conducted and clearly presented, as no statistical tests appear to have been applied.
Reply: We could not perform a time-course analysis of drug efficacy due to constraints intrinsic to the YFP-based functional assay and to the very low activities measured for this CFTR variants. However, the time points chosen for the experiments are the most robust and commonly used (48 h post-transfection, 24 h drug treatment) for this type of analysis. As requested by the reviewer, we are now including in the figure the statistical analysis of the data.

Figure 6E: Only a scatter plot of the data is shown. Representative traces from the corresponding experiments should also be provided to support the quantitative data.
Reply: According to reviewer's comment, we have modified the figure (now revised Fig. 5) to include also representative traces recorded for patient FI066.

Short-circuit current recording (CF-HNE): The transepithelial resistance (TER) values should be reported to validate the quality and consistency of the epithelial monolayers used.
Reply: As requested, we are now reporting, in the Material and Methods section, the data on the transepithelial resistance (TER) values to validate the quality and consistency of the epithelial monolayers used.

Methods – HS-YFP: The authors state that halide-sensitive YFP was used, but the specific YFP variant is not clearly described. Detailed information on the YFP construct (e.g., mutation sites or reference to the original plasmid) should be included for reproducibility.
Reply: We have added this information in the Material and Methods, Cell Culture section, including the citation to the original report that describes the YFP variant and provides information on the plasmid.

Reviewer #2: This is an interesting paper manuscript, detailing that the CFTRdele2ins182 variant, previously considered a null allele, is capable of producing a defective CFTR protein, potentially responsive to modulator rescue therapy. The laboratory work is detailed and well explained.
Reply: We thank the reviewer for the generous praise.

IRCCS – Istituto Giannina Gaslini
Via Gerolamo Gaslini, 5 16147 Genova – Italia
tel. + 39 010 5636 1 | www.gaslini.org
